# Cat scratch disease unveils hidden breast carcinoma: A diagnostic twist

**DOI:** 10.1016/j.radcr.2024.05.075

**Published:** 2024-06-18

**Authors:** Marina Balbino, Manuela Montatore, Federica Masino, Francesca Anna Carpagnano, Giuseppe Guglielmi

**Affiliations:** a Department of Clinical and Experimental Medicine, Foggia University School of Medicine, Viale L. Pinto 1, 71122 Foggia, (FG) Italy; b Breast Unit, Dimiccoli” Hospital, Viale Ippocrate 15, 70051, Barletta (BT), Italy; c Radiology Unit, “Dimiccoli” Hospital, Viale Ippocrate 15, 70051, Barletta (BT), Italy; d Radiology Unit, “IRCCS Casa Sollievo della Sofferenza” Hospital, Viale Cappuccini 1,71013 San Giovanni Rotondo, (FG) Italy

**Keywords:** Cat scratch disease, Bartonella henselae, Breast cancer, Carcinoma in situ, Lymph nodes, Mammography

## Abstract

Cat scratch disease is a rare condition that can present with different clinical manifestations, including axillary lymphadenopathy. Here, we report the case of a 45-year-old female who presented with axillary lymphadenopathy attributable to a process of differential diagnosis to cat scratch disease.

During the thorough investigation of her condition, a routine mammogram was performed, due to the unilateral axillary lymphadenopathy, revealing the presence of previously undiagnosed breast carcinoma in situ; in fact, a DCIS (invasive ductal carcinoma with spread to the ipsilateral axillary nodes) was incidentally found.

This case highlights the importance of comprehensive differential diagnosis and a multidisciplinary approach in managing patients with atypical presentations of common diseases, given that other alarming but unrelated findings are visible.

## Introduction

Cat scratch disease is an infection caused by Bartonella Henselae, commonly affecting young adults. It presents with a range of clinical manifestations, including fever, lymphadenopathy, and sometimes cutaneous lesions at the site of inoculation [1,2]. This case is focused on the importance of the differential and workup of unilateral axillary lymphadenopathy.

Unilateral axillary lymphadenopathy is one of the most common presentations. Breast carcinoma in situ is an early form of breast cancer that has not yet spread beyond the ducts of the mammary gland; it is indeed challenging to recognize in its early forms, particularly on mammographic imaging. Detecting carcinoma in situ requires significant expertise and experience in interpreting mammograms [3].

The patient was fortunate that a more commonplace reason like lymphadenopathy led to the discovery of cancer and it's important to be sure that there are no other unrelated suspicious findings.

## Case report

**Fig. 1:**
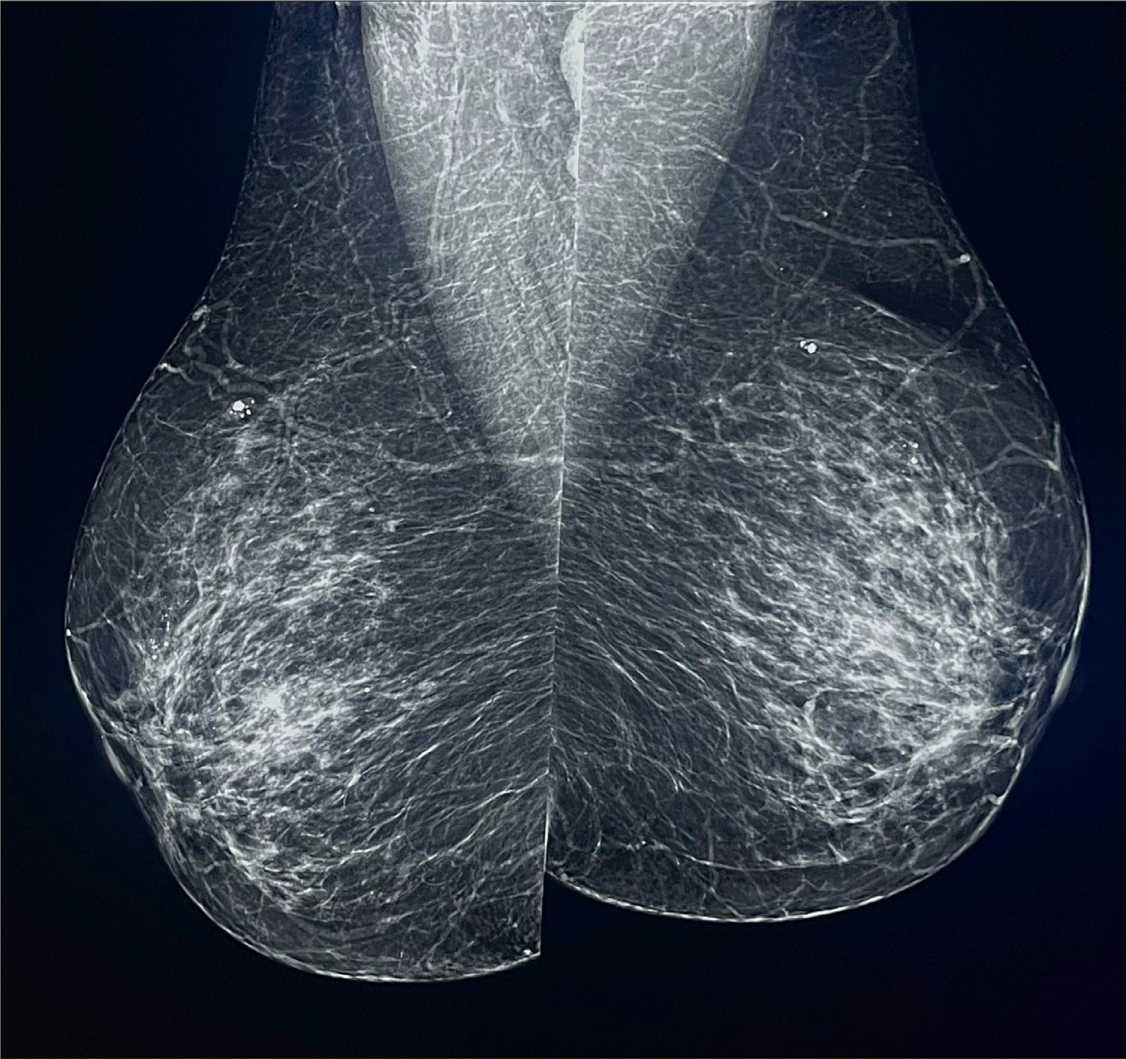
Mammography images, mediolateral-oblique projection: in the upper retro-areolar region of the right breast, there were fine linear microcalcifications. In the right axilla, radiopaque oval-shaped images were observed.

The patient was a 45-year-old woman with no significant past medical history. She has no known family history of cancer or other relevant conditions. Additionally, she was a nonsmoker and did not report any other detrimental health habits. She reported having contact with a stray cat in the weeks prior and had several scratches on her arms as evidence, some of which were deep [5]. The patient presented to our clinic with a history of a painful palpable finding in the right axilla and intermittent fever. Upon physical examination, right axillary lymphadenopathy was confirmed. Using diagnostic mammography and ultrasonography, the unilateral palpable finding in the right axilla was worked up following ACR guidelines and extent and ruled out any other breast or axillary abnormalities (Fig. 1).

**Fig. 2:**
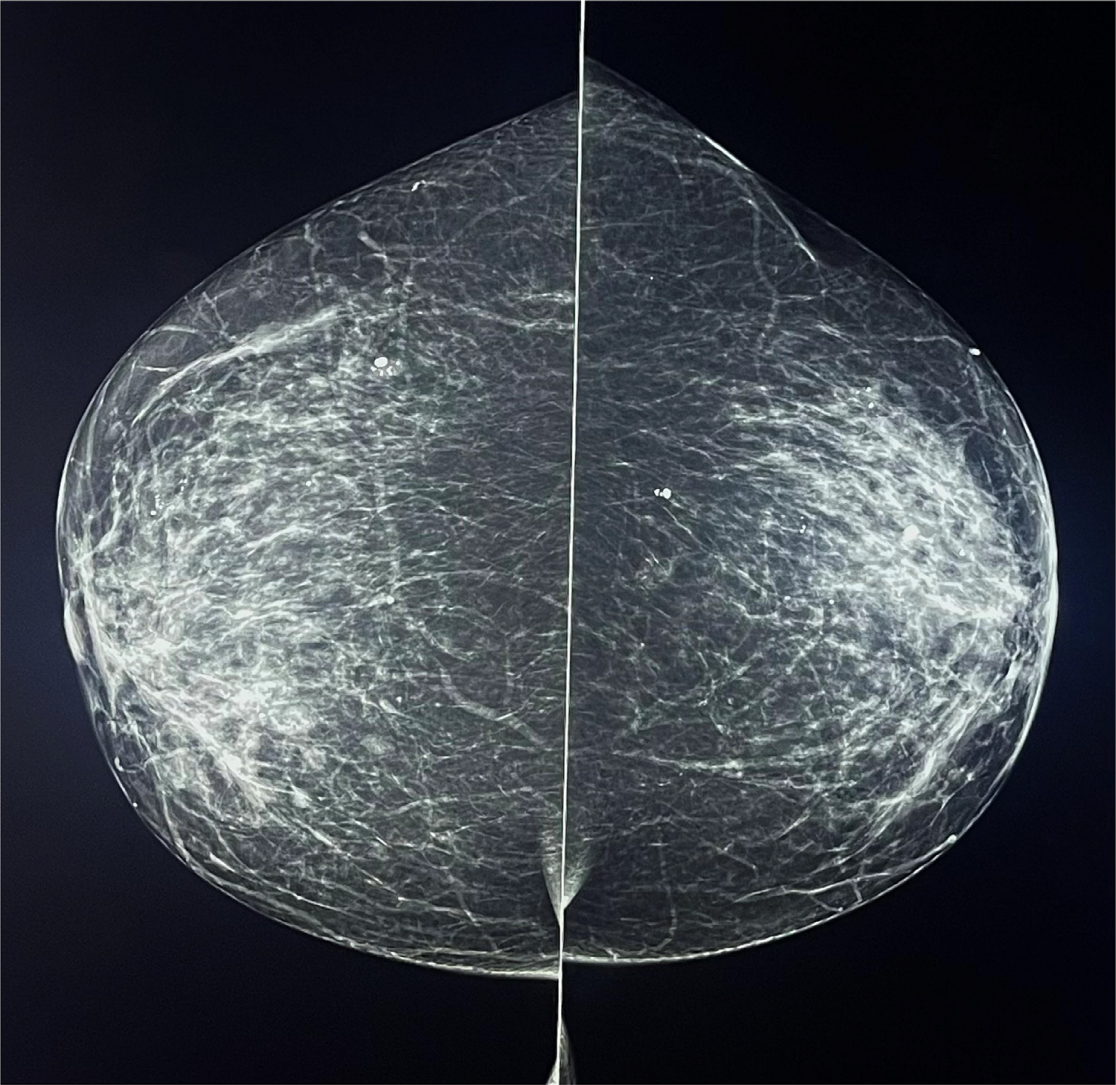
Mammography images, craniocaudal projection: in the outer retro-areolar area of the right breast, there were fine linear microcalcifications.

The patient underwent a routine mammogram, which revealed, within the context of fibro-glandular breast tissue (ACR-B), an area of suspicious microcalcifications in the retro-areolar region of the right breast. Additionally, radiopaque images along the axillary column were only visible in the oblique projection, likely indicative of enlarged lymph nodes. No distortion or suspicious image was identifiable in the other quadrants or the contralateral breast (Fig. 2).

**Fig. 3:**
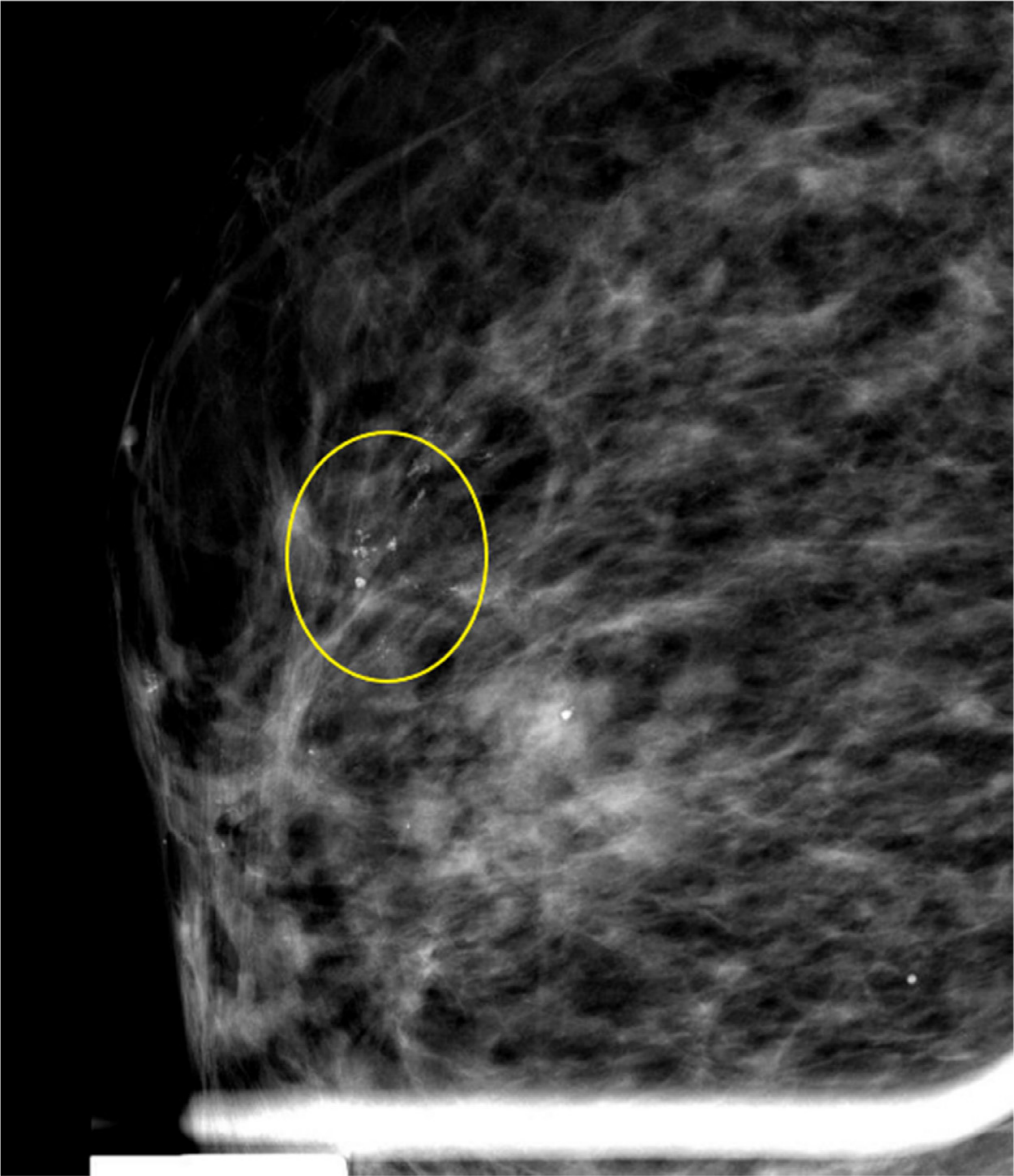
Mammographic magnification: Fine linear microcalcifications were visible in the retro-areolar area of the right breast.

Furthermore, magnification of the area in the retro-areolar region of the right breast was performed to better visualize the morphology and extent of the microcalcifications. The microcalcifications appeared finely linear within an area with a total extent of approximately 2 cm (Fig. 3).

**Fig. 4:**
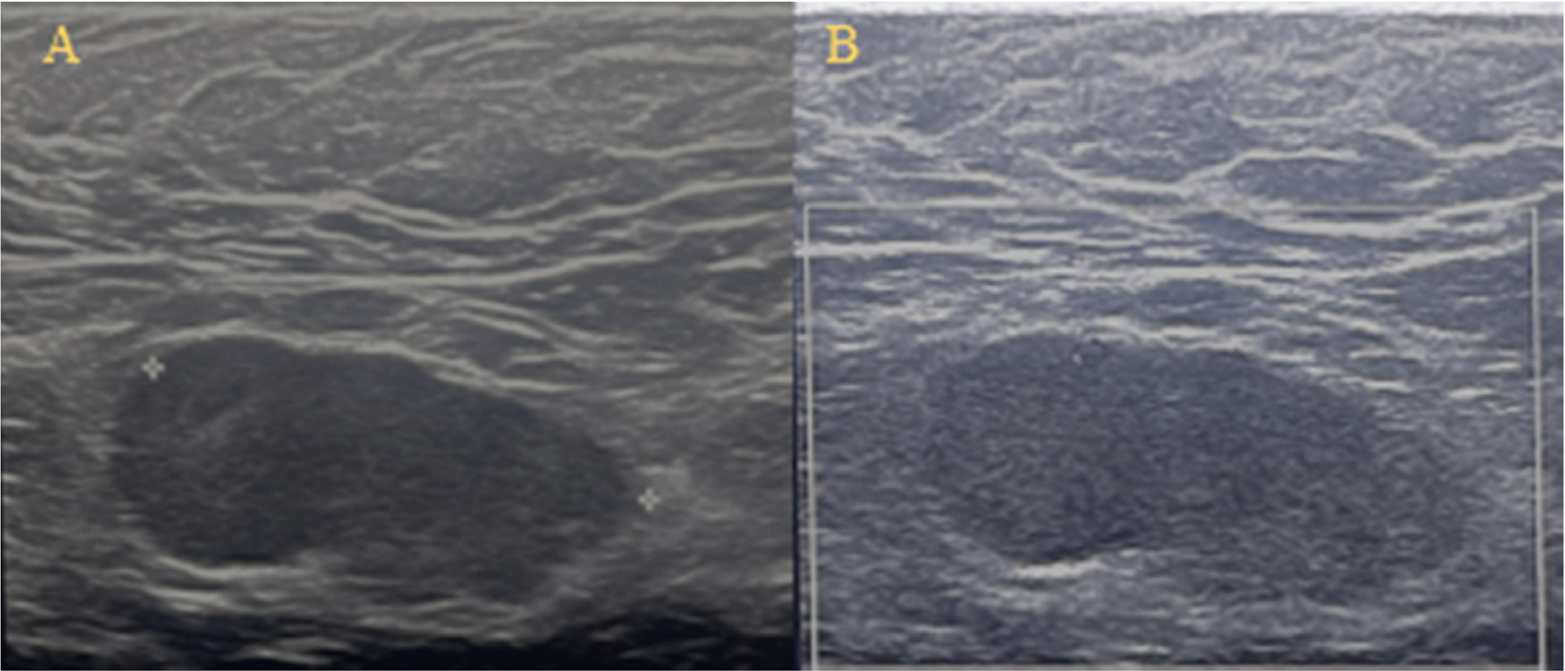
Ultrasound. Irregularly hypoechoic oval-shaped formation in the right axilla (measured about 2.89 cm) (A); No significant increase in vascularization was observed on colour Doppler ultrasound (B).

Subsequently, the patient underwent an ultrasound (Fig. 4).

The lymph nodes in the axilla appeared enlarged, markedly hypoechoic compared to the surrounding tissues, with a thickened cortex and no visible hilum. At least 3 lymph nodes were involved, the largest measuring just under 3 cm in size. These sonographic characteristics are suspicious and indicative of inflammation, infection, or malignancy. However, no suspicious images were found in the breast tissue.

A biopsy was deemed necessary because the sonographic characteristics of the lymph nodes—enlarged size, markedly hypoechoic appearance compared to surrounding tissues, thickened cortex, and absence of a visible hilum—are suspicious and indicative of potential underlying conditions such as inflammation, infection, or malignancy. Despite the lack of a significant increase in vascularization on color Doppler ultrasound, these findings warrant further investigation to determine the exact nature of the lymph node abnormalities and to guide appropriate treatment.

For the lymph nodes, an ultrasound-guided biopsy with a 14G TruCut needle was scheduled. For the area of suspicious microcalcifications, a stereotactic VABB biopsy with a 9G needle was scheduled [6], [7], [8].

An MRI was also scheduled suspecting a possible axillary lymph node involvement (CUP syndrome).

CUP syndrome stands for “Cancer of Unknown Primary”. It refers to a situation where cancer has spread to distant sites in the body (such as lymph nodes) but the primary site where the cancer originated is unknown. This condition poses a diagnostic challenge because identifying the primary tumor site is crucial for determining the most effective treatment strategy. The MRI was scheduled suspecting possible axillary lymph node involvement as part of the evaluation for CUP syndrome. In cases where there are enlarged lymph nodes with suspicious characteristics, but no clear primary tumor is identified, additional imaging tests such as MRI may be performed to help locate the primary tumor or further characterize the extent of the disease. The MRI was performed before receiving the histological diagnosis to expedite the diagnostic process and gather additional information about the extent of the disease.

Finally, the MRI results were completely negative for the breasts; no suspicious areas were visualized after the contrast injection. While not the primary imaging modality for lymph nodes, the MRI still revealed the presence of suspicious nodes, enlarged, and showing marked contrast enhancement.

The results of an image-guided biopsy revealed the presence of cat scratch disease in the right axillary lymph nodes and ductal carcinoma in situ (DCIS), a non-invasive form of breast cancer centered around microcalcifications in the breast.

After the biopsy results, the patient underwent consultations with breast surgeons and oncologists to discuss treatment options based on the diagnosis of DCIS. Treatment for DCIS typically involves surgical excision, and in this case, a quadrantectomy was performed. The final histopathology confirmed ductal carcinoma in situ with positive estrogen and progesterone hormone receptor status. Subsequently, the patient underwent radiation therapy and hormonal therapy. After treatment, the patient would undergo regular follow-up visits with healthcare providers to monitor for any signs of recurrence or complications. For the cat scratch disease affecting the axillary lymph nodes, appropriate management with antibiotics (azithromycin) was initiated to treat the infection. The patient also received education and support regarding cat scratch disease, including lifestyle modifications and strategies for overall health and well-being. The discovery of breast carcinoma in situ alongside cat scratch disease highlights the importance of thorough evaluation and multidisciplinary care in managing patients with complex medical conditions. Each condition requires specific interventions tailored to its characteristics and implications for the patient's health and well-being.

## Discussion

The simultaneous discovery of cat scratch disease and breast carcinoma in situ in this patient represents a rare and intriguing clinical scenario. While axillary lymphadenopathy is a well-known manifestation of cat scratch disease, its presence in this case prompted a diagnostic mammographic workup which led to the unexpected diagnosis of breast carcinoma [1], [2], [3], [4], [5].

This case highlights the importance of thorough evaluations in patients presenting with common or straightforward symptoms, as incidental findings of significant conditions, such as DCIS, can occur and require prompt intervention.

In clinical practice, clinicians must maintain a high index of suspicion and pursue appropriate diagnostic pathways when encountering unusual or unexpected clinical features. In this instance, the discovery of axillary lymphadenopathy during an evaluation for cat scratch disease triggered further investigations, including mammography and ultrasound, ultimately leading to the identification of breast carcinoma [9,10].

The differential diagnosis for unilateral enlarged axillary lymph nodes with thickened cortex involves considering several possible underlying causes [3].

The specific diagnosis requires a comprehensive evaluation, including medical history, physical examination, imaging studies, and sometimes biopsy of the affected lymph nodes [11,12]. The underlying cause of enlarged axillary lymph nodes can vary widely, ranging from benign reactive processes to serious conditions like lymphoma or metastatic cancer.

• **Reactive Lymphadenopathy**:
  ○ **Infection**: Common causes include infections (e.g., cellulitis, abscess).
• **Malignant Lymphadenopathy**:
  ○ **Lymphoma**: Hodgkin lymphoma or non-Hodgkin lymphoma can cause enlarged lymph nodes with cortical thickening.
  ○ **Metastatic Cancer**: Breast cancer, lung cancer, melanoma, or other solid tumors can spread to the axillary lymph nodes.

The differential diagnosis for breast microcalcifications involves considering various benign and malignant conditions that can present with this imaging finding on mammography. Microcalcifications are tiny calcium deposits within the breast tissue that can be indicative of different underlying processes.

1. **Benign lesions**:
  • **Benign Breast Calcifications**: These can be related to benign changes in breast tissue, such as fibroadenomas, fibrocystic changes, or adenosis. Benign calcifications are often coarse, irregular, and scattered throughout the breast tissue (popcorn calcifications).
  • **Calcifications from Previous Trauma or Surgery**: Calcifications can sometimes occur as a result of previous breast surgery, trauma, or inflammation. They may appear as irregular calcifications associated with the previously affected area.
  • **Post-treatment Changes**: Calcifications can sometimes occur as a result of radiation therapy or other treatments for breast cancer. They may appear as irregular and scattered calcifications.
2. **Pre-malignant Lesions**:
  • **Atypical Ductal Hyperplasia (ADH)**: ADH is a non-invasive proliferation of abnormal cells within the breast ducts, which can sometimes present with microcalcifications. These calcifications can be irregular and scattered.
3. **Malignant lesions (These calcifications may appear as fine linear and concentrated)**:
  • **Ductal Carcinoma in Situ (DCIS)**: DCIS is a non-invasive breast cancer confined to the milk ducts, and it often manifests as microcalcifications on mammography.
  • **Lobular Carcinoma in Situ (LCIS)**: LCIS can occasionally present with calcifications.
  • **Invasive Ductal Carcinoma (IDC)**: More advanced breast cancer that has invaded beyond the ducts can also present with calcifications.

The collaborative nature of modern healthcare is indispensable when dealing with intricate cases like this one. For example, in the case of cat scratch disease, it's crucial to understand its presentation and treatment. Similarly, in addressing ductal carcinoma in situ (DCIS), a focused approach involving lumpectomy, radiation therapy, and endocrine therapy for hormone-positive cases is key. While collaboration among primary care physicians, radiologists, oncologists, and other specialists remains vital, tailoring interventions to specific conditions enhances patient care and outcomes. Additionally, this case highlights the value of patient education and awareness. Encouraging patients to communicate openly about their symptoms and medical history, including recent exposures or activities, can provide valuable clues that guide the diagnostic process [13].

In summary, this case serves as a reminder of the unpredictable nature of medicine and the importance of remaining vigilant, curious, and thorough in clinical practice [14,15]. By maintaining a comprehensive and open-minded approach to patient care, healthcare providers can better identify and address underlying serious conditions, ultimately improving patient outcomes and quality of life.

## Conclusion

The case underscores the pivotal role of a comprehensive approach to differential diagnosis and the importance of a multidisciplinary team in managing patients with unusual clinical presentations. Specifically, in this case, the differential diagnosis suggested that ductal carcinoma in situ (DCIS) was incidentally discovered due to its consideration within the broader scope of possible conditions, such as invasive ductal carcinoma (IDC).

Although cat scratch disease is often thought to be benign, this case shows how it can reveal more serious underlying disorders, such in situ breast cancer. Healthcare professionals need to keep an open mind while making differential diagnoses, particularly in the event of unexpected results or symptoms. The identification of incidental breast cancer in this instance was made possible by additional study beyond the standard course of cat scratch disease due to the presence of axillary lymphadenopathy. In conclusion, this case highlights the unpredictability of medicine and the significance of maintaining vigilance, curiosity, and thoroughness in clinical practice. Healthcare professionals can more effectively detect and treat underlying significant illnesses by keeping an all-encompassing and flexible approach to patient care, which will eventually improve patient outcomes and quality of life.

## Patient consent

Complete written informed consent was obtained from the patient for the publication of this study and accompanying images.
